# An effective drug-disease associations prediction model based on graphic representation learning over multi-biomolecular network

**DOI:** 10.1186/s12859-021-04553-2

**Published:** 2022-01-04

**Authors:** Hanjing Jiang, Yabing Huang

**Affiliations:** 1 Key Laboratory of Image Information Processing and Intelligent Control of Education Ministry of China, Institute of Artificial Intelligence, School of Artificial Intelligence and Automation, Huazhong University of Science and Technology, Wuhan, 430074 China; 2 Department of Pathology, Renmin Hospital of Wuhan University, Wuhan, 430060 Hubei China

**Keywords:** Drug-disease association, Graph representation learning, Multi-biomolecular network

## Abstract

**Background:**

Drug-disease associations (DDAs) can provide important information for exploring the potential efficacy of drugs. However, up to now, there are still few DDAs verified by experiments. Previous evidence indicates that the combination of information would be conducive to the discovery of new DDAs. How to integrate different biological data sources and identify the most effective drugs for a certain disease based on drug-disease coupled mechanisms is still a challenging problem.

**Results:**

In this paper, we proposed a novel computation model for DDA predictions based on graph representation learning over multi-biomolecular network (GRLMN). More specifically, we firstly constructed a large-scale molecular association network (MAN) by integrating the associations among drugs, diseases, proteins, miRNAs, and lncRNAs. Then, a graph embedding model was used to learn vector representations for all drugs and diseases in MAN. Finally, the combined features were fed to a random forest (RF) model to predict new DDAs. The proposed model was evaluated on the SCMFDD-S data set using five-fold cross-validation. Experiment results showed that GRLMN model was very accurate with the area under the ROC curve (AUC) of 87.9%, which outperformed all previous works in terms of both accuracy and AUC in benchmark dataset. To further verify the high performance of GRLMN, we carried out two case studies for two common diseases. As a result, in the ranking of drugs that were predicted to be related to certain diseases (such as kidney disease and fever), 15 of the top 20 drugs have been experimentally confirmed.

**Conclusions:**

The experimental results show that our model has good performance in the prediction of DDA. GRLMN is an effective prioritization tool for screening the reliable DDAs for follow-up studies concerning their participation in drug reposition.

## Introduction

Drugs can relieve the symptoms of illness, control the further development of the disease, and help the body to recover. Owning to the increasingly abrupt outbreak of diseases, the demand for new drugs is also on the rise. For example, the sudden outbreak of COVID-19 requires researchers to develop drugs and vaccines in a short period of time. Drug repositioning can effectively reduce the cost of drug development by more than half. Although many researchers have proposed some models for predicting drug-disease associations for drug reposition, how to effectively extract drug-disease association information is still a challenging problem. Analyzing the complex association between drugs and diseases from the microscopic perspective of biomolecules in cells can provide new insights for exploring the mechanism of disease.

Through the integration of large-scale genomic and protein data, a network model is constructed. This provides new ideas for predicting the association between disease molecules and drug molecules. The emergence of network-based predictive approaches not only comprehensively synthesizes associations among protein, miRNA, lncRNA, diseases, and drugs, but also provides a promising computational tool for determining new DDAs and repositioning drugs.

There have been many studies on predicting drug repositioning, including some network-based models. For example, Yu et al*.* proposed to use Layer Attention Graph Convolutional Network (LAGCN) to predict DDA, which use the graph convolution to learn DDA, drug-drug similarity and disease-disease similarity, and use the attention mechanism to combine multiple graph convolutions layers [1]. SCMFDD is a DDA prediction method based on matrix factorization, which maps drug-disease associations into low-rank space and introduces disease semantic similarity and drug similarity increase constraints [2]. Zhang et al. used a binary network to predict DDAs, selecting only drugs and disease information [3]. Researchers are gradually solving the computational problem of drug repositioning from a macro perspective, but previous studies of DDA prediction have not considered the whole cell. The FSPGA algorithm proposed by He et al*.* can effectively detect more meaningful clustering hidden in the attribute graph, taking into account the topology structure and attribute value of the graph [4]. CCPMVFGC proposed by He et al*.* which can well capture the contextual interdependency of features in each cluster by combining graph clustering with multi-view learning [5]. The MrSBM model proposed by He et al. performs unsupervised learning tasks in network data. In addition to modeling edges located within blocks or connecting blocks, MrSBM also considers modeling vertex features using vertex-clustering preferences and probability of feature-clustering contributions [6].

**Fig. 1:**
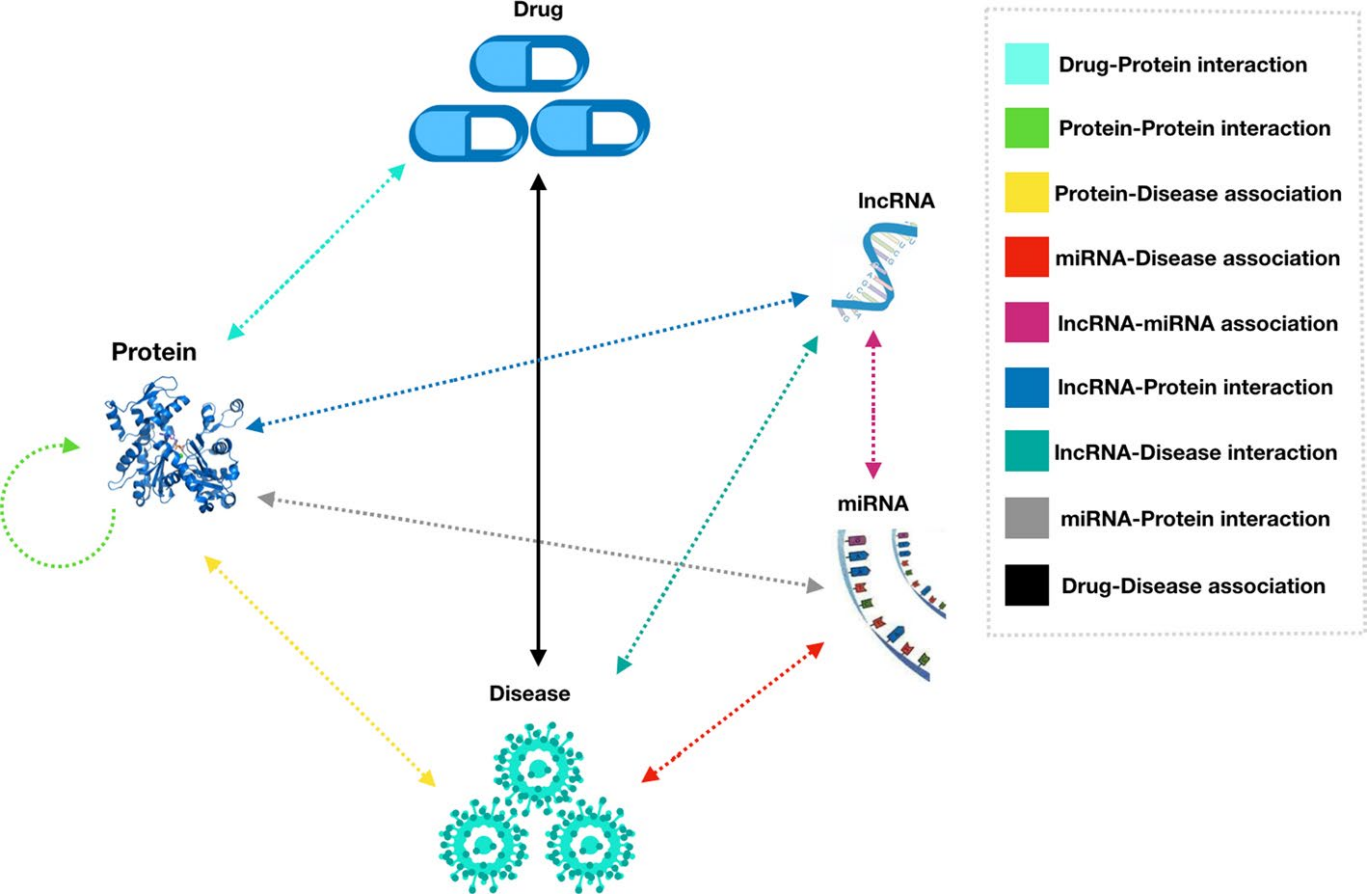
Schematic diagram of the complex relationship among miRNA, lncRNA, protein, disease, and drugs

In previous studies on DDA, some have considered adding an "intermediate bridge" molecule (such as miRNA and protein) between drugs and diseases [7]. With regard to this idea of adding intermediate biomolecules to search for DDA, whether adding more types of biomolecules and the following higher complexity of the MAN network will guarantee a better effect of DDA prediction? In fact, the combination of two biomolecules is a complicated law, and it is not the case that a better DDA prediction effect can be assured with the increase of the number of the intermediate biomolecules. If multiple types of biomolecules data are introduced into the DDA prediction model, most of them will be equivalent to noise, which will directly affect the prediction results. Based on the previous studies of miRNA-disease associations, lncRNA-disease associations, drug-protein associations, and disease-protein associations, we have designed a DDA prediction model that uses protein, lncRNA, and miRNA as intermediate molecules. As shown in Fig. 1, there are 9 confirmed associations among the five biomolecules [8].

**Fig. 2:**
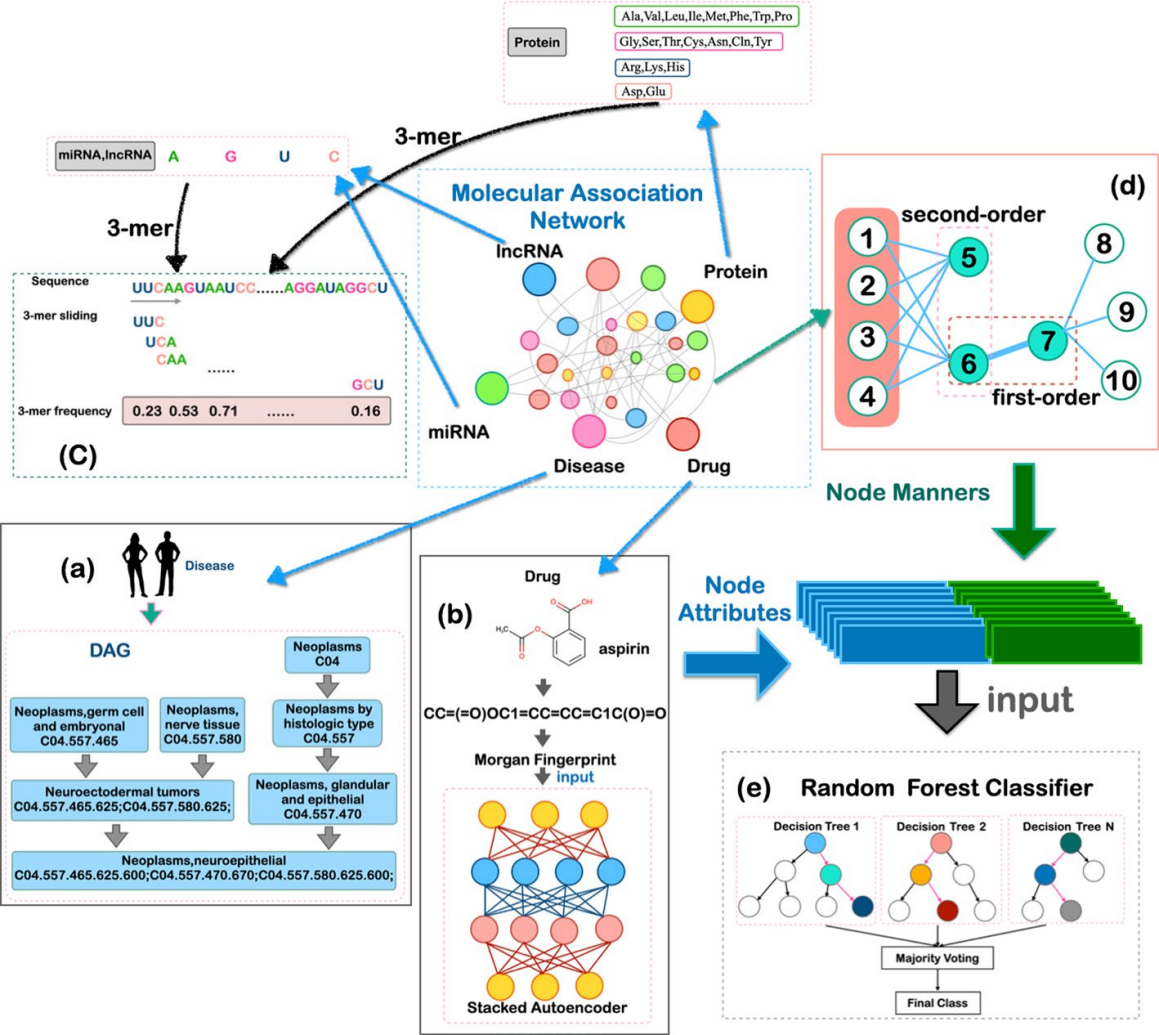
MAN is formed by associations between proteins, miRNAs, lncRNAs, drugs, and diseases. The attributes and behaviors of biomolecules learned by graph embedding are input into random forest classifier for training to predict potential drug-disease associations. **a** process of computing semantic similarity of diseases by constructing directed acyclic graph; **b** process of obtaining the Morgan fingerprint and extracting the structural similarity of the drug; **c** 3-mer analysis of the sequence; **d** a schematic diagram of LINE; **e** prediction process of random forest

Graphs are one of the most powerful framework in algorithms, and can be used to represent almost all types of structures or systems. Different biomolecules and their interactions can be viewed as vertices (nodes) and links (edges) in a graph [9]. Based on the above, in this paper, we constructed a molecular association network (MAN), including miRNA, lncRNA, protein, drug, disease, and nine associations (lncRNA-protein interaction [10], drug-protein association [11], protein–protein interaction [12, 13], protein-disease interaction [14], miRNA-disease association [15], miRNA-disease association [16], miRNA-lncRNA association [17], lncRNA-disease interaction [18], and drug-disease association [19]). Each node in the MAN is composed of the attribute of the node itself and the associated information with other nodes. Node information includes drug molecular fingerprint, disease semantic information, ncRNA sequence, and protein sequence [20]. A unique feature of GRLMN combines five biomolecules and nine molecular associations [21]. Although this paper mainly solves the problem of drug repositioning, GRLMN has better scalability and can predict the association between other molecules using the proposed network model [22]. Figure 2 shows the workflow of GRLMN model, in which the complex network of biomolecules consists of two parts: nodes (drug, disease, protein, miRNA, and lncRNA) and edges (the relationship of nodes) [23].

To evaluate the ability of the GRLMN to predict DDAs, fivefold cross-validation method was performed on SCMFDD-S data set [24]. Through the comparison with different feature models and classifier models, the proposed model achieved good results [25]. In addition, we also tested the validity of the model for two human diseases, including Kidney disease and Fever [26]. As a result, among the top 20 drugs predicted by GRLMN that are related to kidney disease or fever, 15 have been verified in the comparative toxicogenomics database (CTD) [27]. Experiment results show that the proposed model combines node attribute information and mode information to obtain effective robust prediction performance [28]. Complex molecular association networks allow us to understand biology and disease pathology from a global perspective.

## Materials and methods

### Multi-biomolecular associations data

In this work, the SCMFDD-S data set collected by Zhang et al*.* [29] is used for training, which includes 269 drugs, 598 diseases, and 18,416 DDAs. DrugBank [30] is a comprehensive database of extensive drug information, providing SMILE for drugs. We use python packages to convert SMILE to Morgan fingerprints. In addition, as shown in Table 1, we downloaded eight types of heterogeneous associations from nine other databases, 8374 pairs of miRNA-lncRNA association provided by lncRNASNP2 database, 16,427 pairs of miRNA-disease association provided by HMDD database [31], 4944 pairs of miRNA-protein association provided by miRTarBase database [32], and 1264 pairs of lncRNA-disease association provided by LncRNADisease [33] and lncRNASNP2 [34] databases. LncRNA2Target [35], DisGeNET [36], DrugBank, and STRING [37] provided 690 pairs of lncRNA-protein associations, 25,087 pairs of protein-disease associations, 11,107 pairs of drug-protein associations, and 19,237 pairs of protein–protein interactions [38–40]. After unifying identifiers, eliminating redundancy, simplify, and deleting irrelevant items, the downloaded experimental data are sorted out and obtained in Table 2.

**Table 1:** Details of nine kinds of biomolecular association used by the proposed model

Association type	Database	Number of associations
Drug-disease	SCMFDD-S [29]	18,416
Drug-protein	DrugBank [30]	11,107
Protein–protein	STRING [37]	19,237
Protein-disease	DisGeNET [36]	25,087
lncRNA-protein	LncRNA2Target [35]	690
lncRNA-disease	LncRNADisease [33] lncRNASNP2 [34]	1264
miRNA-protein	miRTarBase [32]	4944
miRNA-disease	HMDD [31]	16,427
miRNA-lncRNA	lncRNASNP2 [34]	8374
Total		105,546

**Table 2:** The number of five types of nodes in the proposed model

Node	Drug	Disease	LncRNA	MiRNA	Protein	Total
Number	1025	2062	769	1023	1649	6528

### Disease descriptors

In order to represent the similarity between diseases, we calculated disease semantic similarity by referring to the MeSH database [41], which developed by the National Library of Medicine (NLM). The MeSH database categorizes diseases strictly and accurately. Each disease we download from https://www.nlm.nih.gov/ has a descriptor that can construct a directed acyclic graph (DAG) to describe the disease. Specifically, for disease $e$, and its DAG can be described as ${DAG}_{e}=(e,{N}_{e},{D}_{e})$, where ${N}_{e}$ represents the set of diseases associated with disease $e$, and ${D}_{e}$ represents the set of edges between them. The contribution of a certain disease $d$ to the semantic value of disease $e$ in the set ${N}_{e}$ is:

1 $$\left\{\begin{array}{ll}{C}_{e}\left(d\right)=1 ,& if \,d=e,\\ {C}_{e}\left(d\right)=\mathit{max}\left\{\varepsilon \cdot {C}_{e}\left({\acute{d}}\right)|{\acute{d}}\in children\; of\; d\right\},& if \;d \ne e,\end{array}\right.$$

where $\varepsilon$ is a contribution parameter. The semantic value $DV(e)$ can be obtained by adding up the contribute values of all diseases in the disease set ${N}_{e}$, and its formula is as follows [42]:

2 $$DV\left(e\right)={\sum }_{d\in {N}_{e}}{C}_{e}(d)$$

Assume that the more DAGs shared by two diseases, the more similar they are. Based on this assumption, diseases semantic similarity is calculated according to the relative positions of diseases $e(i)$ and $e(j)$:

3 $${SV}_{1}\left(e\left(i\right),e\left(j\right)\right)=\frac{{\sum }_{d\in {N}_{e\left(i\right)}\bigcap {N}_{e(j)}}({C}_{e\left(i\right)}\left(d\right)+{C}_{e\left(j\right)}(d))}{DV\left(e\left(i\right)\right)+DV(e(j))}$$

### NcRNA and protein sequence descriptors

In order to standardize and characterize the ncRNA transcription and protein sequences, we use 3-mer to analyze each sequence. As shown in Fig. 2, in order to facilitate the coding of proteins and ncRNA, we divided the 20 amino acids and the four nucleotides into 4 groups. The grouping of amino acids is: [Ala, Val, Leu, Ile, Met, Phe, Trp, and Pro], [Gly, Ser, Thr, Cys, Asn, Gln, and Tyr], [Arg, Lys, and His], and [Asp and Glu] [43]. The grouping of ncRNAs is adenine (A), cytosine (C), guanine (G), and uracil (U). As shown in Fig. 2c, we calculate the frequency of each different amino acid or RNA combination through a sliding window of length 3. Here, we can express a 64 (4^3^) dimensional vector through 3-mer.

### Stacked auto-encoder

**Fig. 3:**
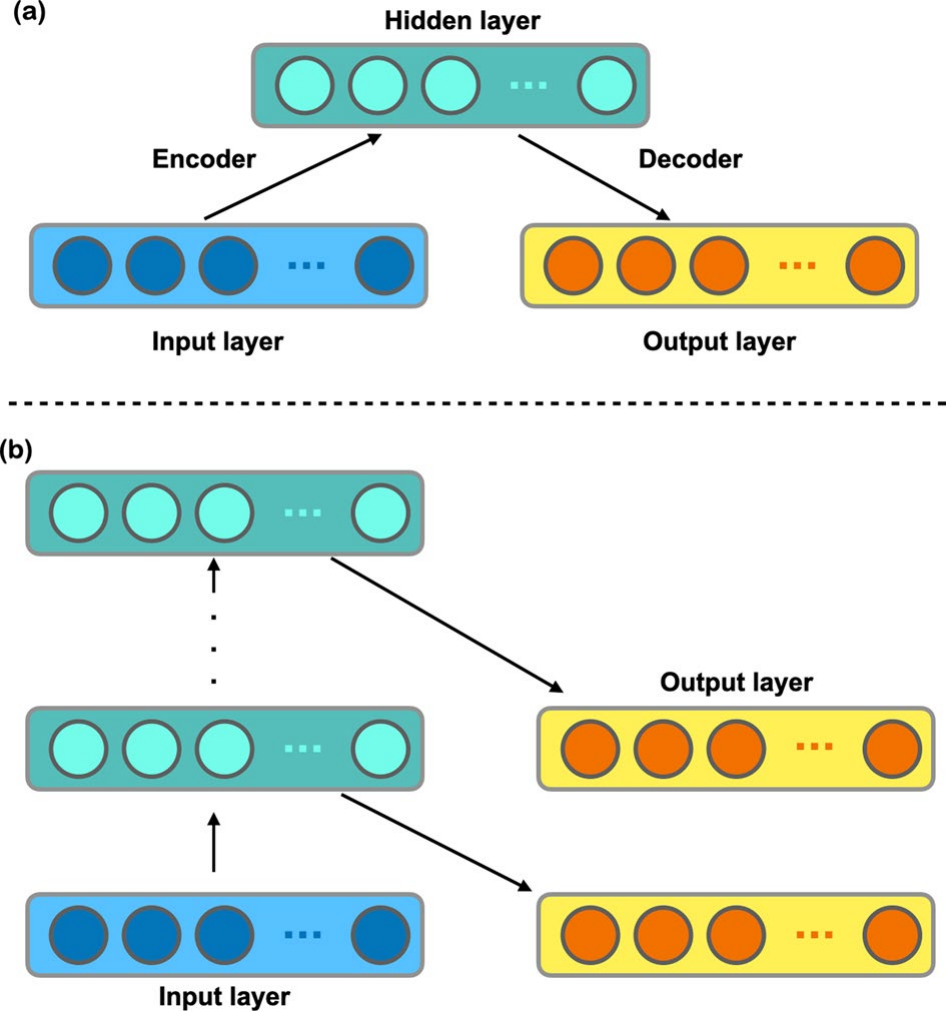
The structure of the auto-encoder and structure of the stacked auto-encoder

As shown in Fig. 2b, the SIMLES (simplified molecular input line entry specification) of the drug can be found in the DrugBank database. The RDkit python package can convert SIMLES into Morgan fingerprints [44, 45]. In this work, Stacked Auto-encoder (SAE) is introduced to extract the constructed Morgan fingerprints. As shown in Fig. 3a, auto-encoder is a kind of symmetric neural network, which belongs to semi-supervised learning, and its learning function is ${\acute{x}}= {f}_{W,b}(x)\approx x$, where $x$ is the input vector, $W=({W}_{1},{W}_{2})$ and $b=({b}_{1},{b}_{2})$ represent the weights and biases.

Figure 3b shows the structure of a stacked auto-encoder with an h-stage auto-encoder. The vector output by the first auto-encoder layer is used as the vector of the second auto-encoder layer input until the output vector of the top autoencoder layer is obtained. The random gradient descent was selected for training. Drug molecular fingerprints obtain a vector characterizing molecular structure by stacking autoencoder.

### Node representation

In the MAN, each node is composed of two parts, one is the attributes of the node itself, and the other is the association with other nodes. Attributes of the node itself include ncRNA sequences, protein sequences, semantic information of disease, and drug fingerprints. Specifically, the network representation learning is used to calculate the association between nodes and other nodes which can globally represent the information flow between the entire network nodes. Due to the sparseness and discreteness of the MAN network, we urgently need a simple and efficient low-dimensional representation method to represent it, and graph embedding is such a method. As the current mainstream network embedding algorithm, LINE [46] can embed large-scale information networks into low-order vector spaces and is suitable for any type of information network.

LINE is a method based on the assumption of neighborhood similarity, which can be seen as an algorithm that use Breath First Search (BFS) to construct neighborhoods. A major feature of LINE is that it optimizes the goal of preserving local nodes and global network structure. LINE combines the first-order similarity and second-order similarity in the graph structure to obtain richer graph representation results. Figure 2d explains first-order and second-order. The thickness of the edge represents the value of the weight. Because node 6 and node 7 are directly connected and have a larger weight, their first-order similarity is higher. In the MAN network, the weights of the edges are all equal. Node 5 and node 6 are not directly connected, but they share a common adjacent node, so their embedding should have a similar distance and a greater second-order similarity.

First-order is to model each undirected edge. First, calculate the probability distribution of node transition. For each directed edge $(a,b)$, we first define the probability that the neighbor of vertex ${v}_{a}$ is ${v}_{b}$ as:

4 $${p}_{1}\left({v}_{b}|{v}_{a}\right)=\frac{1}{1+\mathrm{exp}(-{u}_{b}^{T}\cdot {u}_{a})} ,$$

where ${u}_{a}$ and ${u}_{b}$ are the embedding vector representations of node $a$ and node $b$, respectively. According to the weights of the edges, the empirical distribution can also be obtained:

5 $${{p}_{1}}^{\prime}\left(a,b\right)=\frac{{\omega }_{ab}}{W} ,W= {\sum }_{i,j\epsilon E}{\omega }_{ij},$$

where $W$ is the sum of the weights of the edges in the graph. In order to keep the empirical distribution similar to the probability distribution, we use KL divergence to measure the similarity of the two distributions. After we remove the constant term, the loss function obtained is as follows:

6 $${L}_{1}= -{\sum }_{\left(a,b\right)\epsilon E}{\omega }_{ab}\mathrm{log}\left({p}_{1}\left({v}_{a},{v}_{b}\right)\right),$$

therefore, as long as the ${L}_{1}$ is minimized, we can guarantee the first-order similarity of node embedding in the graph.

Second-order applies to both directed and undirected graphs. We first define the probability distribution of node transition:

7 $${p}_{2}\left({v}_{b}|{v}_{a}\right)=\frac{\mathrm{exp}({{\acute{u}}_{b}}^{T}\cdot {u}_{a})}{{\sum }_{k=1}^{|V|}\mathrm{exp}({{\acute{u}}_{k}}^{T}\cdot {u}_{a})}$$

where $\left|V\right|$ is the number of vertices, ${u}_{a}$ is the representation when ${v}_{a}$ is regarded as vertex and ${{\acute{u}}_{a}}$ is the representation of ${v}_{a}$ when it is treated as a specific “context”. At the same time, the second-order empirical distribution is defined as follows:

8 $${{p}_{2}}^{\prime}\left({v}_{b}|{v}_{a}\right)=\frac{{\omega }_{ab}}{{d}_{a}} ,{d}_{a}= {\sum }_{k\in N(i)}{\omega }_{ik},$$

where ${d}_{a}$ is the output degree of node $a$ and $N(i)$ is the adjacent node of node $i$.

To make sure the empirical distribution and the probability distribution similar. we use KL divergence to measure the similarity of the two distributions. After removing the constant term and performing a series of approximations, we get the loss function as follows:

9 $${L}_{2}= -{\sum }_{\left(a,b\right)\epsilon E}{\omega }_{ab}\mathrm{log}\left({p}_{2}\left({v}_{b}|{v}_{a}\right)\right).$$

### Random forest

Ensemble learning has been widely used in bioinformatics, the idea of which is to combine multiple single classifiers into a new classifier to obtain better classification effect. We choose the random forest classifier in the ensemble learning algorithm to classify and predict the drug-disease association [47]. Random forest can avoid the problem of decision tree overfitting. Compared with other single classifiers, it usually has more stable prediction performance [48]. Since stability and accuracy are very important for large-scale prediction of drugs-diseases association, in this work, random forest was selected as the classifier to process the extracted features.

## Results and discussion

### Evaluation criteria

In order to verify the prediction ability of GRLMN, fivefold cross-validation method was performed on the real data set collected in Table 1 in the experiment. Specifically, fivefold cross-validation is to randomly divide the sample into 5 subsets of the same number. Each time a subset is selected as the test set, and the remaining subsets are used as the training set. The training process is repeated five times so that each subset could be used as the test set, and the average of the five groups is used as the finally result. To quantify the results of fivefold cross-validation, we selected five kinds of evaluation criteria, including sensitivity (SEN), specificity (SPE), precision (PRE) accuracy (ACC) and Matthews correlation coefficient (MCC). The calculation formula is as follows:

10 $$SEN. = \frac{TP}{TP+FN} ,$$

11 $$SPE = \frac{TN}{FP+TN} ,$$

12 $$PRE= \frac{TP}{TP+FP} ,$$

13 $$ACC= \frac{TP+TN}{TP+TN+FP+FN} ,$$

14 $$MCC = \frac{TP\times TN-FP\times FN}{\sqrt{(TP+FP)(TP+FN)(TN+FP)(TN+FN)}} ,$$

where TP is true positive, FP is false positive, TN is true negative and FN is false negative. For further evaluation, we also compute the receiver operating characteristic (ROC) curve, sum up the ROC curve in a numerical way, and calculate the area under the ROC curve (AUC).

### Evaluate prediction performance

In this section, fivefold cross-validation method was performed on the SCMFDD-S data set to evaluate the ability of the proposed model to predict DDAs. Table 3 shows that in the experiment on the SCMFDD-S data set, GRLMN yielded the average accuracy, sensitivity, specificity, and precision of GRLMN are all around 80%, and the Matthews correlation coefficient is 59.68%. In a huge network of nine biomolecule association relationships, all indicators can perform well, which shows that GRLMN has good predictive ability by fusing molecular features.

**Table 3:** fivefold cross-validation results performed by the three models GRLMN, GRLMN_A, and GRLMN_M

Model	ACC (%)	SEN (%)	SPE (%)	PRE (%)	MCC (%)	AUC (%)
GRLMN	79.84 ± 0.50	80.03 ± 0.95	79.64 ± 0.22	79.72 ± 0.28	59.68 ± 1.00	87.90 ± 0.54
GRLMN_A	73.91 ± 0.32	75.43 ± 0.77	72.39 ± 0.34	73.20 ± 0.20	47.84 ± 0.64	81.12 ± 0.28
GRLMN_M	77.58 ± 0.54	78.41 ± 1.07	76.75 ± 0.46	77.14 ± 0.39	55.18 ± 1.10	85.61 ± 0.42

**Fig. 4:**
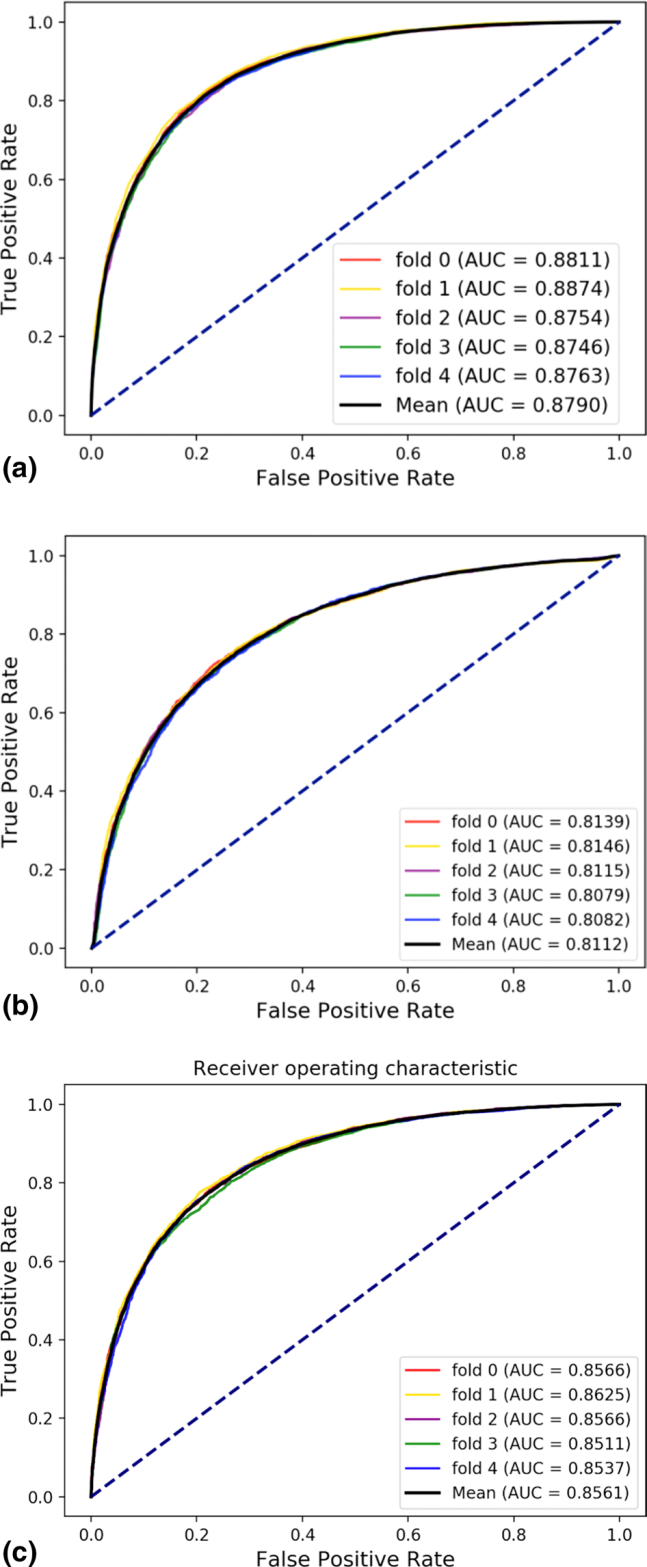
Performance yielded GRLMN in DDA prediction: **a** ROC curves yielded by GRLMN using fivefold cross-validation on SCMFDD-S data set. **b** ROC curves yielded by GRLMN_A using fivefold cross-validation on SCMFDD-S data set. **c** ROC curves yielded by GRLMN_M using fivefold cross-validation on SCMFDD-S data set

As mentioned in “Node representation” section, GRLMN calculates the association between each node and other nodes through LINE algorithm to predict DDA. In this section, we also evaluated the effectiveness of the introduction of node association information and node attribute information. We call the model that only uses the attributes of the node itself as GRLMN_A, and the model that only uses the associated attributes of the node as GRLMN_M. As shown in Table 3 and Fig. 4, without using the node's own attribute features, the prediction performance of GRLMN_M in fivefold cross-validation is significantly reduced, but all indicators are still higher than those in GRLMN_A. The comparison results showed that the attributes of the node itself and the associated attributes of the node in GRLMN were closely related and mutually beneficial to the prediction task.

### Impact of different graph embedding on GRLMN

Graph Embedding has been widely used in recommender systems and computational advertising, and the corresponding algorithms are constantly being extended. In this section, we discuss the difference between applying LINE and Node2vec in the GRLMN model. Node2vec adjusts the weights of random walks to make the results of graph embedding weighed in the homophily and structural equivalence of the network. Specifically, the "homophily" of the network means that the embedding of nodes that are close to each other should be as close as possible, and the "structural equivalence" means that the embedding of nodes that are structurally similar should be as close as possible.

**Fig. 5:**
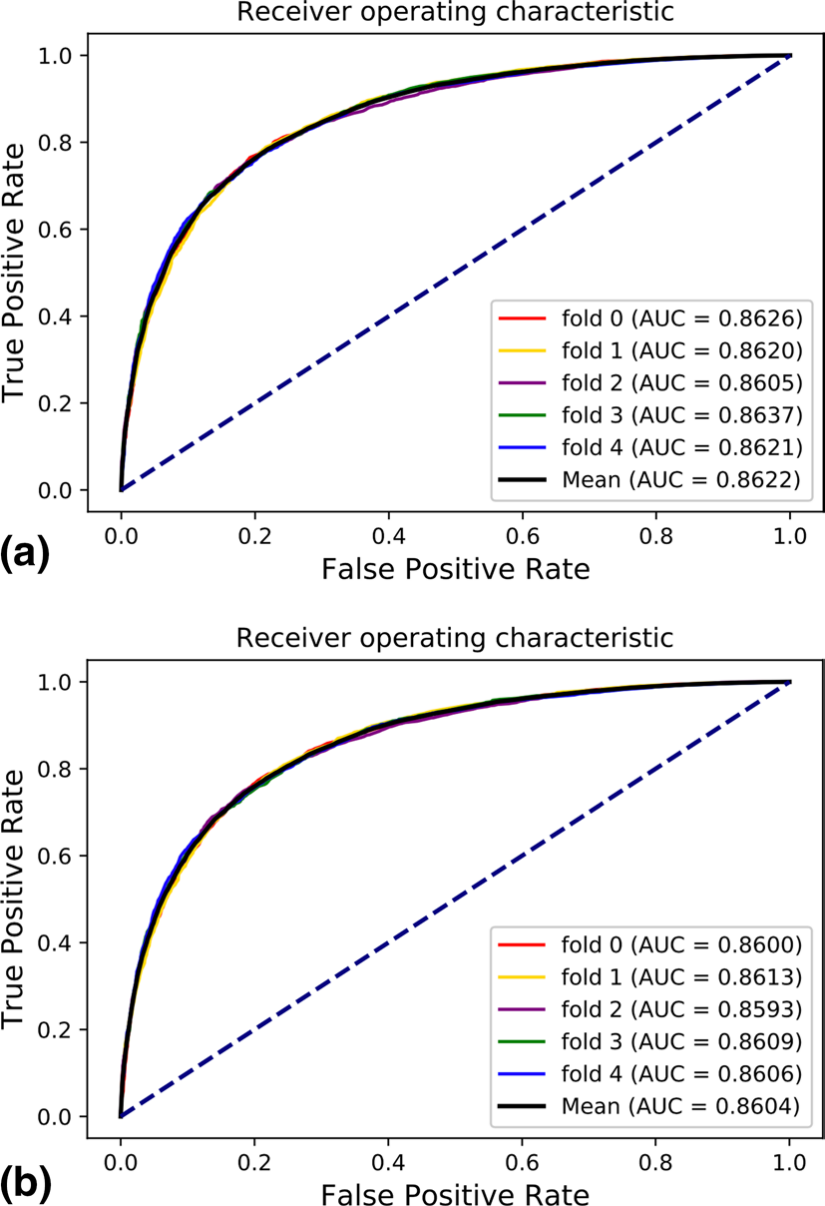
Performance yielded GRLMN-node2vec in DDA prediction: **a** ROC curves yielded by GRLMN-node2vec using fivefold cross-validation on SCMFDD-S data set. **b** ROC curves yielded by GRLMN-node2vec containing only attribute using fivefold cross-validation on SCMFDD-S data set

Based on the control variable method, we replace the LINE part of GRLMN with Node2vec, and the rest remain unchanged. For the sake of distinction, we call GRLMN based on Node2vec as GRLMN-node2vec, and GRLMN based on LINE as GRLMN-LINE. Figure 5a is the fivefold cross-validation AUC curve of GRLMN-node2vec on the SCMFDD-S data set. Figure 5b is the ROC curves yielded by GRLMN-node2vec containing only attribute using fivefold cross-validation on SCMFDD-S data set. The AUC result of GRLMN-node2vec is 0.18% higher than that of GRLMN-node2vec which only contains attribute features, but its performance is still inferior to GRLMN-LINE. LINE is based on the edge sampling algorithm to improve and optimize the objective function, which overcomes the limitations of the traditional stochastic gradient descent algorithm, so the effect will be better.

### Performance comparison

To further verify the performance of GRLMN in predicting DDA, we performed fivefold cross-validation of the other six models on the same data set. SCMFDD model proposed by Zhang et al*.* [29], which proposed mapping the association between drugs and diseases to two low-rank spaces, using matrix decomposition to predict associations. Table 4 shows the average AUC value of the other six models and our method. From the table we can see that GRLMN achieves a higher average AUC value on SCMFDD-S data set. In the SCMFDD-S data set, the AUC obtained by the proposed model was the highest, 0.78% higher than the AUC generated by LNS, 0.53% higher than SCMFDD-Drug interaction, 1.16% higher than SCMFDD-Enzyme, 0.81% higher than SCMFDD-Pathway, 3.77% higher than SCMFDD-Target, and 0.5% higher than SCMFDD-Substructure. The Experimental results show that GRLMN has more advantages. Unlike the comparison method, GRLMN is more extensible, which uses the attribute of five biological molecules and their association to form a molecular association network. We integrate more comprehensive molecular information to achieve significant prediction results.

**Table 4:** Comparison of AUC values generated by different methods on benchmark data set

Methods	AUC (%)
SCMFDD-substructure	87.37
SCMFDD-target	84.10
SCMFDD-pathway	87.06
SCMFDD-enzyme	86.71
SCMFDD-drug interaction	87.34
LNS	87.09
GRLMN	87.87

### Impact of different classifier on GRLMN

GRLMN use random forest to make predictions based on feature fusion. In this section, we evaluate the effectiveness of random forest. Specifically, we use Adaboost classifier, Logistic Regression classifier, and Naïve Bayes classifier to replace of random forest classifier to compare the effectiveness of GRLMN and the combination of these classifiers. According to the control variable method, all kinds of experimental data are the same except for different classifiers. In order to make results more credible, fivefold cross-validations were performed on the four models simultaneously. Use grid search to find the best parameters of random forest: n_estimators = 100, max_depth = 110. Adaboost classifier, Logistic Regression classifier, and Naive Bayes classifier all adopt default parameters.

**Fig. 6:**
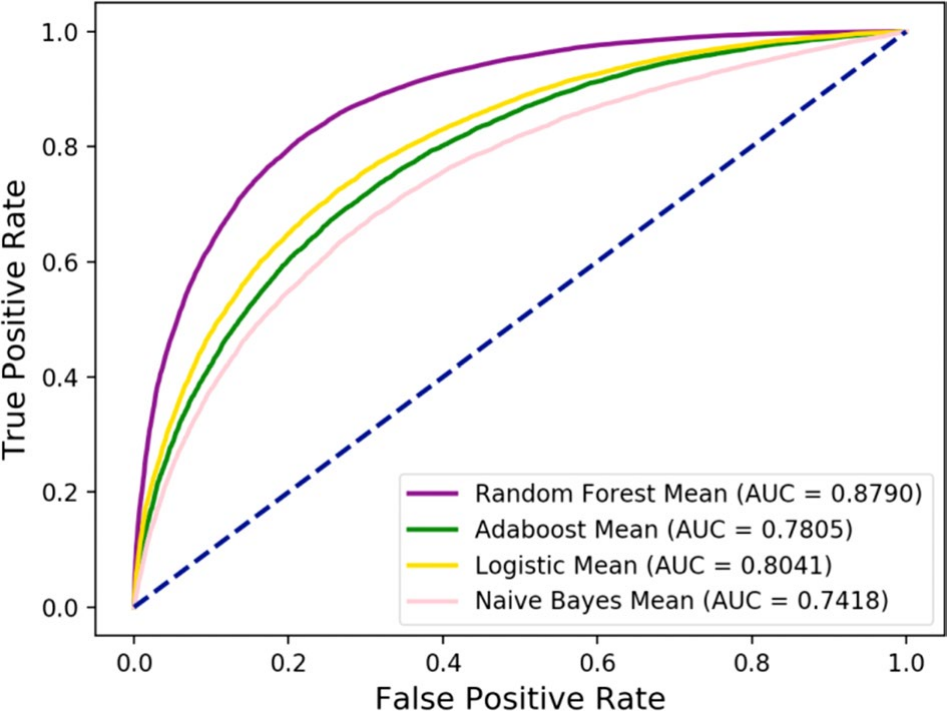
Comparison of AUC values obtained by Random Forest, Adaboost, Logistic Regression, and Naive Bayes classifier models on the same data set

Table 5 and Fig. 6 show the results of combining the random forest classifier, the Adaboost classifier, the Logistic regression classifier, and the Naive Bayes classifier with the proposed feature descriptors. Adaboost classifier achieved accuracy, sensitivity, specificity, precision, MCC, and AUC of 70.82%, 71.30%, 70.34%, 70.62%, 41.65%, and 78.05%, respectively. Their standard deviations are 0.35%, 1.15%, 0.88%, 0.41%, 0.71%, and 0.52%. Logistic regression classifier achieved accuracy, sensitivity, specificity, precision, MCC, and AUC of 72.95%, 72.98%, 72.92%, 72.94%, 45.91%, and 80.41%, respectively. Their standard deviations are 0.45%, 0.99%, 0.68%, 0.44%, 0.91%, and 0.54%. Naïve Bayes classifier achieved accuracy, sensitivity, specificity, precision, MCC and AUC of 68.27%, 70.86%, 65.69%, 67.37%, 36.60%, and 74.18%, respectively. Their standard deviations are 0.55%, 0.86%, 0.76%, 0.53%, 1.10%, and 0.62%. It can be seen from the comparison that the classification results of random forest classifier are superior to the other four classifiers. The average AUC of the random forest is 9.85%, 7.49%, and 13.72% higher than that of Adaboost classifier, Logistic Regression classifier, and Naive Bayes classifier, respectively.

**Table 5:** Comparison of results of different classifier models on the same data set

Classifier	ACC (%)	SEN (%)	SPE (%)	PRE (%)	MCC (%)	AUC (%)
Adaboost	70.82 ± 0.35	71.30 ± 1.15	70.34 ± 0.88	70.62 ± 0.41	41.65 ± 0.71	78.05 ± 0.52
Logistic	72.95 ± 0.45	72.98 ± 0.99	72.92 ± 0.68	72.94 ± 0.44	45.91 ± 0.91	80.41 ± 0.54
Naïve Bayes	68.27 ± 0.55	70.86 ± 0.86	65.69 ± 0.76	67.37 ± 0.53	36.60 ± 1.10	74.18 ± 0.62
Random forest	79.84 ± 0.50	80.03 ± 0.95	79.64 ± 0.22	79.72 ± 0.28	59.68 ± 1.00	87.90 ± 0.54

## Case study

To further evaluate the ability of GRLMN to predict potential associations, we select kidney disease and fever as cases for experiments. Specifically, we use the SCMFDD-S dataset to train the model. When predicting associations for specified diseases, all associations between specified diseases and drugs in the data set are deleted. According to the prediction results of GRLMN, we validated the top 20 drugs with predicted scores in the independent CTD database.

Kidney disease is usually caused by factors such as infection, genetics, and immunity. As shown in Table 6, we validated the top 20 drugs for Kidney disease prediction in the CTD database and identified 15 of them. Fever is a state in which abnormal body temperature or excessive heat production and heat dissipation caused by various reasons, resulting in a rise in body temperature beyond the normal range. The top ranked drugs related to fever predicted by the GRLMN model are listed in Table 7. Comparing the prediction results with the CTD database, 15 of them were confirmed. Associations not listed in the CTD database may actually exist but are not currently verified.

**Table 6:** The proposed model predicted the top 20 drugs associated with Kidney disease

Index	Drug name	Validation result	Index	Drug name	Validation result
1	Carbamazepine	Confirmed	11	Niacin	Confirmed
2	Amiodarone	Confirmed	12	Nicotine	Confirmed
3	Ramipril	Confirmed	13	Quinine	Confirmed
4	Piroxicam	Confirmed	14	Epinephrine	N/A
5	Sulindac	Confirmed	15	Troglitazone	N/A
6	Tretinoin	N/A	16	Procainamide	Confirmed
7	Naproxen	Confirmed	17	Digoxin	Confirmed
8	Docetaxel	N/A	18	Chloroquine	Confirmed
9	Clozapine	Confirmed	19	Norfloxacin	N/A
10	Methyldopa	Confirmed	20	Hydrocortisone	Confirmed

**Table 7:** The proposed model predicted the top 20 drugs associated with Fever disease

Index	Drug name	Validation result	Index	Drug name	Validation result
1	Lidocaine	Confirmed	11	Digoxin	Confirmed
2	Propranolol	N/A	12	Levodopa	Confirmed
3	Diazepam	Confirmed	13	Norepinephrine	Confirmed
4	Fluoxetine	N/A	14	Ribavirin	Confirmed
5	Naloxone	N/A	15	Midazolam	Confirmed
6	Paroxetine	N/A	16	Celecoxib	Confirmed
7	Methadone	Confirmed	17	Hydrocortisone	Confirmed
8	Epinephrine	Confirmed	18	Timolol	N/A
9	Furosemide	Confirmed	19	Naltrexone	Confirmed
10	Ofloxacin	Confirmed	20	Desipramine	Confirmed

## Conclusion

Drug reposition requires a lot of theoretical support from DDA, so it is a meaningful work to develop an algorithm for predicting DDA. In this paper, the association among drug, disease miRNA, lncRNA, and protein were integrated, and the multi-biomolecular network was constructed from the perspective of cells.

In the experimental, we evaluated GRLMN model on SCMFDD-S data set using the fivefold cross-validation method. Experimental results show that the proposed model is highly accurate in predicting drug indications and significantly superior to other methods. In addition, case studies of Kidney disease and Fever have shown that GRLMN has outstanding performance in predicting a list of potential drugs associated with a particular disease. Our prediction model can be applied to the prediction of actual DDA problems. The experimental results show that the large-scale association prediction network based on machine learning model not only supplements the artificial experiment, but also opens up a macroscopic perspective to predict the association between molecules. Similar to the general machine learning framework, there are inevitable disadvantages. When new nodes are added, the network needs to learn the feature again. The addition of new nodes should meet certain conditions: 1. The new node must be linked to the original network and cannot be an isolated node; 2. The more links between new nodes and nodes in the network, better features can be learned; However, the time cost of feature relearning is not very high, and now powerful machine performance can deal with this problem quickly.

## Data Availability

Drug-disease association is downloaded from SCMFDD-S database, drug-protein association is downloaded from DrugBank database, protein–protein interaction is downloaded from STRING database, and protein-disease association is downloaded from DisGeNET database. lncRNA-protein associations are downloaded from the LncRNA2Target database. The lncRNA -disease association is downloaded from the LncRNADisease database and the lncRNASNP2 database. The miRNA-protein association was downloaded from the miRTarBase database, the miRNA-disease association was downloaded from the HMDD database, and the miRNA- lncRNA association was downloaded from the lncRNASNP2 database. Source code of our models and training/testing datasets are available at: https://github.com/HanJingJiang/GRLMN.

## Abbreviations

DDAs: Drug-disease associations
MAN: Molecular association network
RF: Random forest
SEN: Sensitivity
CTD: The comparative toxicogenomics database
SPE: Specificity
PRE: Precision
ACC: Accuracy
MCC: Matthews correlation coefficient
TP: True positive
FP: False positive
TN: True negative
FN: False negative
ROC: Receiver operating characteristic curve
AUC: The area under the ROC curve
SIMLES: Simplified molecular input line entry specification

## References

[1] Yu Z, Huang F, Zhao X, Xiao W, Zhang W (2020). Predicting drug–disease associations through layer attention graph convolutional network. Brief Bioinform.

[2] Zhang W, Yue X, Lin W, Wu W, Liu R, Huang F, Liu F (2018). Predicting drug-disease associations by using similarity constrained matrix factorization. BMC Bioinform.

[3] Zhang W, Yue X, Chen Y, Lin W, Li B, Liu F, Li X. Predicting drug-disease associations based on the known association bipartite network. In: 2017 IEEE international conference on bioinformatics and biomedicine (BIBM): 2017: IEEE; 2017, p. 503–9.

[4] He T, Chan KC (2018). Discovering fuzzy structural patterns for graph analytics. IEEE Trans Fuzzy Syst.

[5] He T, Liu Y, Ko TH, Chan KCC, Ong Y. Contextual correlation preserving multiview featured graph clustering. IEEE Trans Syst Man Cybern 2019:1–14. 10.1109/TCYB.2019.2926431 31329151

[6] He T, Bai L, Ong Y-S. Manifold regularized stochastic block model. In: 2019 IEEE 31st international conference on tools with artificial intelligence (ICTAI): 2019: IEEE; 2019, p. 800–7.

[7] Yang M, Luo H, Li Y, Wang J (2019). Drug repositioning based on bounded nuclear norm regularization. Bioinformatics.

[8] Yue X, Wang Z, Huang J, Parthasarathy S, Moosavinasab S, Huang Y, Lin SM, Zhang W, Zhang P, Sun H (2019). Graph embedding on biomedical networks: methods, applications and evaluations. Bioinformatics.

[9] Yi H-C, You Z-H, Huang D-S, Guo Z-H, Chan KCC, Li Y (2020). Learning representations to predict intermolecular interactions on large-scale heterogeneous molecular association network. iScience.

[10] Yi H-C, You Z-H, Huang D-S, Li X, Jiang T-H, Li L-P (2018). A deep learning framework for robust and accurate prediction of ncRNA-protein interactions using evolutionary information. Mol Ther Nucleic Acids.

[11] Li Z, Han P, You ZH, Li X, Zhang Y, Yu H, Nie R, Chen X (2017). In silico prediction of drug-target interaction networks based on drug chemical structure and protein sequences. Sci Rep.

[12] Chen Z-H, Li L-P, He Z, Zhou J-R, Li Y, Wong L (2019). An improved deep forest model for predicting self-interacting proteins from protein sequence using wavelet transformation. Front Genet.

[13] Wang L, Wang H-F, Liu S-R, Yan X, Song K-J (2019). Predicting protein-protein interactions from matrix-based protein sequence using convolution neural network and feature-selective rotation forest. Sci Rep.

[14] Lee HS, Bae T, Lee J-H, Kim DG, Oh YS, Jang Y, Kim J-T, Lee J-J, Innocenti A, Supuran CT (2012). Rational drug repositioning guided by an integrated pharmacological network of protein, disease and drug. BMC Syst Biol.

[15] Zheng K, You Z-H, Wang L, Zhou Y, Li L-P, Li Z-W (2019). MLMDA: a machine learning approach to predict and validate MicroRNA–disease associations by integrating of heterogenous information sources. J Transl Med.

[16] Wang L, You Z-H, Chen X, Li Y-M, Dong Y-N, Li L-P, Zheng K (2019). LMTRDA: Using logistic model tree to predict MiRNA-disease associations by fusing multi-source information of sequences and similarities. PLoS Comput Biol.

[17] Huang Z-A, Huang Y-A, You Z-H, Zhu Z, Sun Y (2018). Novel link prediction for large-scale miRNA-lncRNA interaction network in a bipartite graph. BMC Med Genom.

[18] Chen X (2015). Predicting lncRNA-disease associations and constructing lncRNA functional similarity network based on the information of miRNA. Sci Rep.

[19] Wong L, You ZH, Ming Z, Li J, Chen X, Huang YA (2015). Detection of interactions between proteins through rotation forest and local phase quantization descriptors. Int J Mol Sci.

[20] Wang Y, You Z, Li L, Chen Z (2020). A survey of current trends in computational predictions of protein-protein interactions. Front Comp Sci.

[21] Guo Z-H, You Z-H, Huang D-S, Yi H-C, Zheng K, Chen Z-H, Wang Y-B (2021). MeSHHeading2vec: a new method for representing MeSH headings as vectors based on graph embedding algorithm. Brief Bioinform.

[22] Guo Z-H, You Z-H, Huang D-S, Yi H-C, Chen Z-H, Wang Y-B (2020). A learning based framework for diverse biomolecule relationship prediction in molecular association network. Commun Biol.

[23] Zheng K, You Z-H, Wang L, Zhou Y, Li L-P, Li Z-W (2020). Dbmda: A unified embedding for sequence-based mirna similarity measure with applications to predict and validate mirna-disease associations. Mol Ther Nucleic Acids.

[24] Jiang H-J, You Z-H, Zheng K, Chen Z-H. Predicting of drug-disease associations via sparse auto-encoder-based rotation forest. In: International conference on intelligent computing: 2019: Springer; 2019, p. 369–80.

[25] Jiang H-J, Huang Y-A, You Z-H (2020). SAEROF: an ensemble approach for large-scale drug-disease association prediction by incorporating rotation forest and sparse autoencoder deep neural network. Sci Rep.

[26] Wang Y-B, You Z-H, Yang S, Yi H-C, Chen Z-H, Zheng K (2020). A deep learning-based method for drug-target interaction prediction based on long short-term memory neural network. BMC Med Inform Decis Mak.

[27] Jiang H-J, You Z-H, Huang Y-A (2019). Predicting drug− disease associations via sigmoid kernel-based convolutional neural networks. J Transl Med.

[28] Wong L, You Z-H, Guo Z-H, Yi H-C, Chen Z-H, Cao M-Y. MIPDH: a novel computational model for predicting microRNA–mRNA interactions by DeepWalk on a heterogeneous network. ACS Omega. 2020. 10.1021/acsomega.9b04195 PMC7376568 32715187

[29] Zhang W, Yue X, Lin W, Wu W, Liu R, Huang F, Liu F (2018). Predicting drug-disease associations by using similarity constrained matrix factorization. BMC Bioinform.

[30] Wishart DS, Feunang YD, Guo AC, Lo EJ, Marcu A, Grant JR, Sajed T, Johnson D, Li C, Sayeeda Z (2018). DrugBank 5.0: a major update to the DrugBank database for 2018. Nucleic Acids Res.

[31] Li Y, Qiu C, Tu J, Geng B, Yang J, Jiang T, Cui Q (2014). HMDD v2.0: a database for experimentally supported human microRNA and disease associations. Nucleic Acids Res.

[32] Chou C-H, Shrestha S, Yang C-D, Chang N-W, Lin Y-L, Liao K-W, Huang W-C, Sun T-H, Tu S-J, Lee W-H (2018). miRTarBase update 2018: a resource for experimentally validated microRNA-target interactions. Nucleic Acids Res.

[33] Chen G, Wang Z, Wang D, Qiu C, Liu M, Chen X, Zhang Q, Yan G, Cui Q (2012). LncRNADisease: a database for long-non-coding RNA-associated diseases. Nucleic Acids Res.

[34] Miao Y-R, Liu W, Zhang Q, Guo A-Y (2018). lncRNASNP2: an updated database of functional SNPs and mutations in human and mouse lncRNAs. Nucleic Acids Res.

[35] Jiang Q, Wang J, Wu X, Ma R, Zhang T, Jin S, Han Z, Tan R, Peng J, Liu G (2015). LncRNA2Target: a database for differentially expressed genes after lncRNA knockdown or overexpression. Nucleic Acids Res.

[36] Piñero J, Bravo À, Queralt-Rosinach N, Gutiérrez-Sacristán A, Deu-Pons J, Centeno E, García-García J, Sanz F, Furlong LI. DisGeNET: a comprehensive platform integrating information on human disease-associated genes and variants. Nucleic Acids Res. 2016:gkw943. 10.1093/nar/gkw943 PMC5210640 27924018

[37] Szklarczyk D, Morris JH, Cook H, Kuhn M, Wyder S, Simonovic M, Santos A, Doncheva NT, Roth A, Bork P. The STRING database in 2017: quality-controlled protein–protein association networks, made broadly accessible. Nucleic Acids Res. 2016:gkw937. 10.1093/nar/gkw937 PMC5210637 27924014

[38] Chen Z-H, You Z-H, Li L-P, Wang Y-B, Wong L, Yi H-C (2019). Prediction of self-interacting proteins from protein sequence information based on random projection model and fast Fourier transform. Int J Mol Sci.

[39] Li Y, Li L-P, Wang L, Yu C-Q, Wang Z, You Z-H (2019). An ensemble classifier to predict protein–protein interactions by combining PSSM-based evolutionary information with local binary pattern model. Int J Mol Sci.

[40] Chen Z-H, You Z-H, Li L-P, Wang Y-B, Li X. RP-FIRF: prediction of self-interacting proteins using random projection classifier combining with finite impulse response filter. In: International conference on intelligent computing: 2018: Springer; 2018, p. 232–40.

[41] Wang D, Wang J, Lu M, Song F, Cui Q (2010). Inferring the human microRNA functional similarity and functional network based on microRNA-associated diseases. Bioinformatics.

[42] Wang L, You Z-H, Chen X, Li Y-M, Dong Y-N, Li L-P, Zheng K (2019). MTRDA: Using logistic model tree to predict miRNA-disease associations by fusing multi-source information of sequences and similarities. PLOS Comput Biol.

[43] Shen J, Zhang J, Luo X, Zhu W, Yu K, Chen K, Li Y, Jiang H (2007). Predicting protein-protein interactions based only on sequences information. Proc Natl Acad Sci USA.

[44] Landrum G. Rdkit documentation. Release. 2013:1–79.

[45] Weininger D (1988). SMILES, a chemical language and information system. 1. Introduction to methodology and encoding rules. J Chem Inf Comput Sci.

[46] Tang J, Qu M, Wang M, Zhang M, Yan J, Mei QJ. LINE: Large-scale information network embedding. 2015:1067–1077.

[47] Breiman L (2001). Random forests. Mach Learn.

[48] Jiang H-J, Huang Y-A, You Z-H (2019). Predicting drug-disease associations via using Gaussian interaction profile and kernel-based autoencoder. Biomed Res Int.

